# Molecular tumor board in advanced sarcoma: treatment implementation and clinical outcomes in a real-world cohort

**DOI:** 10.1007/s00432-026-06651-1

**Published:** 2026-10-22

**Authors:** Christoph Weinfurtner, Moritz Kaupp-Merkle, Ralf Marienfeld, Uwe Gerstenmaier, Jakob Niewöhner, Tobias Kolb, Axel Fürstberger, Sonja Dahlum, Paul Herzog, Ambros J. Beer, Regine Mayer-Steinacker, Meinrad Beer, Thomas F. E. Barth, Thomas Seufferlein, Nadine T. Gaisa, Mathias Wittau, Stephan Stilgenbauer, Alexandra von Baer, Markus Schultheiß, Reiner Siebert, Wolfgang Thaiss, Hartmut Döhner, Verena I. Gaidzik

**Affiliations:** 1 Department of Internal Medicine III, University Hospital Ulm, Albert-Einstein-Allee 23, 89081 Ulm, Germany; 2 Department of Nuclear Medicine, University Hospital Ulm, Ulm, Germany; 3 Institute of Pathology, University Ulm, Ulm, Germany; 4 Institute of Human Genetics, University Hospital Ulm and Ulm University, Ulm, Germany; 5 Comprehensive Cancer Center Ulm, University Hospital Ulm and Ulm University, Ulm, Germany; 6 Department of Diagnostic and Interventional Radiology, University Hospital Ulm, Ulm, Germany; 7 Department of Internal Medicine I, University Hospital Ulm, Ulm, Germany; 8 Center for Personalized Medicine, University Hospital Ulm, Ulm, Germany; 9 Department of General and Visceral Surgery, University Hospital Ulm, Ulm, Germany; 10 Department of Trauma Surgery, Hand Surgery, Plastic Surgery and Reconstructive Surgery, University Hospital Ulm, Ulm, Germany; 11 Center for Rare Diseases Ulm, University Hospital Ulm and Ulm University, Ulm, Germany

**Keywords:** Sarcoma, Molecular tumor board, Precision oncology, Homologous recombination repair, PARP inhibitor, Targeted therapy

## Abstract

**Purpose:**

Advanced sarcomas remain therapeutically challenging. We evaluated treatment implementation and clinical outcomes after molecular tumor board recommendations.

**Methods:**

We retrospectively analyzed 125 sarcoma patients discussed in our multidisciplinary Molecular and Familial Tumor Board between 2017 and 2023. Clinical benefit was assessed by treatment implementation and by the ratio of progression-free survival on molecularly guided therapy versus the preceding treatment line (PFS2/PFS1). Overall survival (OS) was measured from first board discussion.

**Results:**

A total of 252 pathogenic or likely pathogenic variant records across 133 genes were identified. Molecular profiling enabled therapy recommendations in 71 of 125 patients (56.8%), with treatment implementation in 41 (32.8%). PFS2/PFS1 evaluation was feasible in 29 patients, and nine (31.0%) had a documented ratio > 1.3. Median PFS2 was 102 days (95% CI 63–233). Two of seven patients (28.6%) receiving poly(ADP-ribose) polymerase (PARP) inhibitor-containing regimens had ratios > 1.3. Median OS in 124 patients was 14.6 months (95% CI 11.7–18.5; global log-rank *p* = 0.220).

**Conclusion:**

Molecular tumor board-guided therapy identified treatment options for selected sarcoma patients. Prolonged disease control in individual PARP-treated cases provides a positive signal that warrants prospective evaluation.

**Supplementary Information:**

The online version contains supplementary material available at https://doi.org/10.1007/s00432-026-06651-1.

## Introduction

The exceptional heterogeneity of sarcomas, not only in histology but even more in their genetic, epigenetic, and cellular characteristics, might explain the slow progress in the development of molecularly guided therapeutic approaches (Grünewald et al. 2020). Due to their rarity (< 1% of all malignant neoplasms are soft tissue sarcomas, and approximately 0.2% are bone sarcomas) and their complex management, treatment at reference centres and/or within reference networks is commonly recommended to ensure comprehensive care (Gerrand et al. 2025; Spalato-Ceruso et al. 2024). Anthracycline-based chemotherapy remains a standard for advanced soft tissue sarcoma (Leitlinienprogramm Onkologie 2022; Spalato-Ceruso et al. 2024). Likewise, treatment of advanced bone sarcomas, despite entity-specific multimodal chemotherapeutic regimens, has not led to a major breakthrough in the advanced disease setting, whereas gastrointestinal stromal tumors (GIST) illustrate the potential of targeting a dominant oncogenic driver with tyrosine kinase inhibitors (Napolitano and Vincenzi 2019).

Advances in next-generation sequencing and other omics technologies have improved the molecular characterization of sarcoma subtypes and enabled the identification of potentially actionable alterations (Zou et al. 2022). However, most sarcomas are characterized by complex molecular mechanisms rather than single dominant drivers and generally exhibit a comparatively low tumor mutational burden (Chalmers et al. 2017; The Cancer Genome Atlas Research Network 2017). Molecular profiling may therefore provide clinically relevant information, particularly at disease progression when standard treatment options are limited. In this setting, multidisciplinary molecular tumor boards (MTBs) integrate genomic and other molecular findings with clinical and pathological information to refine diagnoses and inform individualized treatment strategies. MTBs may also facilitate access to molecularly matched clinical trials and promote interdisciplinary decision-making (Horak et al. 2021; Wahida and Kurzrock 2025).

In light of this, we retrospectively investigated all sarcoma patients discussed in our Molecular and Familial Tumor Board (MoFa) from 2017 to 2023 focusing on treatment recommendations, their implementation, and clinical outcomes.

## Materials and methods

### Patient population and study design

**Fig. 1:**
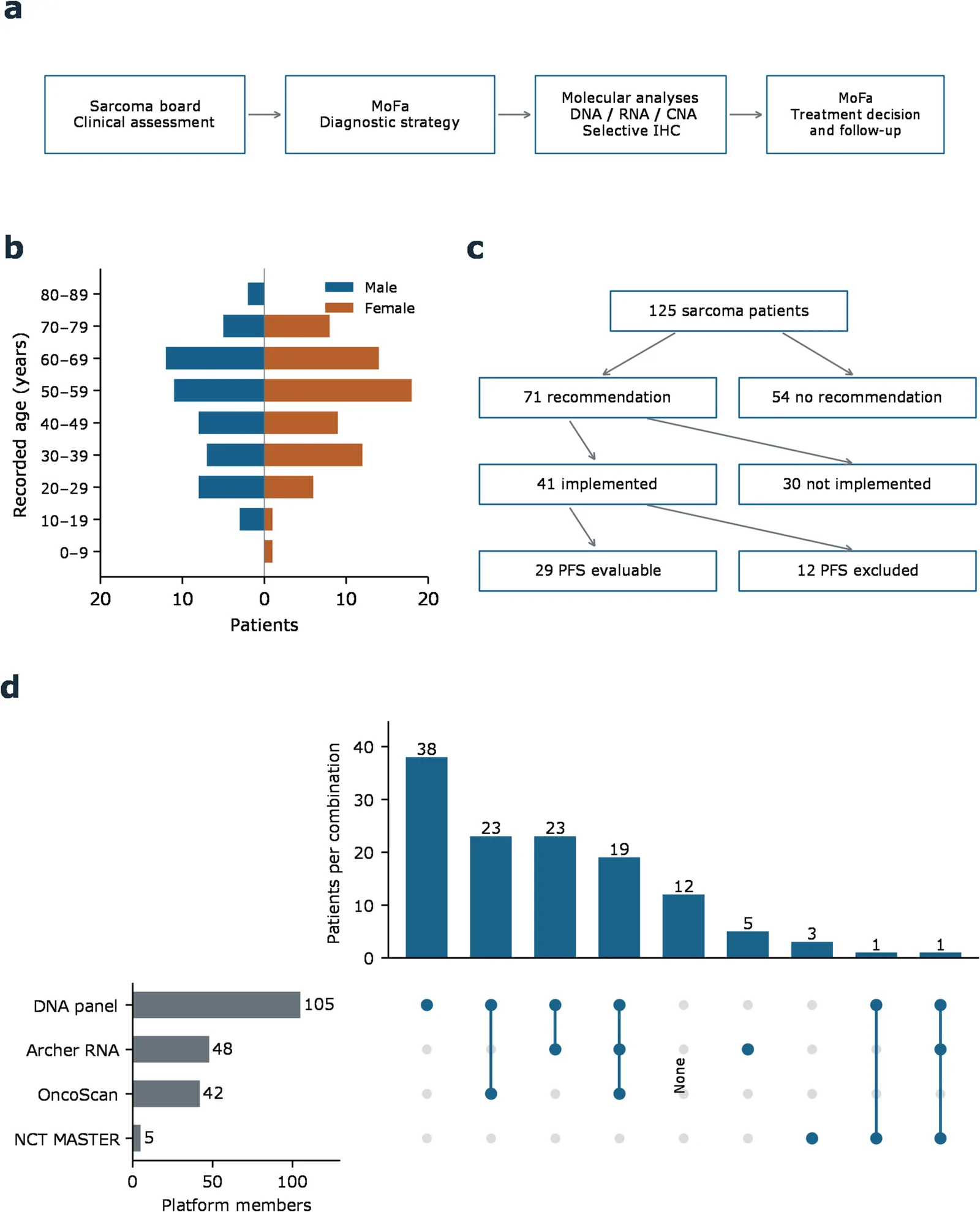
Clinical workflow and cohort. **a**: Sarcoma board and MoFa workflow. **b**: Recorded age by sex. **c**: Cohort of 125 patients; treatment recommendations, *n* = 71, treatment implementations, *n* = 41 and evaluable PFS pairs, *n* = 29. d: Exclusive platform combinations: DNA panel sequencing, *n* = 105, Archer RNA sequencing, *n* = 48, OncoScan analysis, *n* = 42, NCT MASTER profiling, *n* = 5; patients outside all four profiling groups, *n* = 12. RNA counts include documented unsuccessful analyses

All patients with the diagnosis of bone or soft tissue sarcoma (*n* = 125) who were presented in our MTB named “MoFa” between 2017 and 2023 were included, independent of sex, in the retrospective study (Fig. 1a). The MoFa is embedded in our Comprehensive Cancer Center as well as the Center of Personalized Medicine and the Center of Rare Diseases at Ulm University and Ulm University Hospital. One patient did not meet the eligibility criteria and was excluded.

Inclusion criteria for MoFa discussion were (1) advanced disease with limited or exhausted guideline-based therapeutic options, or (2) rare cases without evidence-based guidelines, or (3) genetic tumor syndromes with a need for tumor-specific therapy. Within the framework of our MoFa, decisions were made regarding the specific molecular genetic analyses to be conducted and the sample types to be used.

The research was approved by the local ethics committee of the University of Ulm (references 463/21 and 327/23) and conducted in accordance with the ethical principles outlined in the Declaration of Helsinki (World Medical Association 2025).

### Sample analyses

For tumor-only next-generation sequencing performed at the Institute of Pathology, the following gene panels were used: the 160-gene Human Comprehensive Cancer Panel, the 270-gene Human Comprehensive Cancer Panel V3 and the 486-gene QIAseq Targeted DNA Human Tumor Mutational Burden Panel (Qiagen). Some patients underwent more than one gene panel over time. Additional panels and external molecular analyses were included when available. For RNA-based analysis, Archer FusionPlex Sarcoma, Expanded Sarcoma and Lung panels were used.

Library preparation was done using at least 40 ng of DNA according to the instructions of the manufacturer. Analyses were performed on a MiSeq device (Illumina) with a mean coverage of 2006–4257× and ≥ 100× coverage in 92–99% of target regions. CLC Biomedical Workbench 5.0.1 or CLC Genomics Workbench 12 was used for variant calling. Only class four and five variants classified according to Richards et al. are reported in the Results section (Richards et al. 2015) and are displayed in Supplementary Table S1 (annotated using ANNOVAR) (Wang et al. 2010).

Copy-number determination using OncoScan array analyses (Affymetrix) was performed at the Institute of Human Genetics on tumor DNA and evaluated using the ChAS software. Imbalance profiles were evaluated with regard to potential therapeutic options considering diagnosis, prior therapies and druggable targets, partially taking other molecular findings (e.g. sequencing data) into account. Focal imbalances of < 5 Mb were predominantly considered as indicators of potential therapy targets.

Additionally, genomic profiling data were available via participation in the NCT MASTER program (Molecularly Aided Stratification for Tumor Eradication Research). Comprehensive genomic and transcriptomic profiling was performed in five patients (Horak et al. 2021), counted separately from documented DNA-panel testing.

Platform overlap was assessed at the patient level, with each patient counted once per platform (Fig. 1d). DNA-panel and Archer-RNA membership included documented unsuccessful analyses. The choice of testing depended on tissue availability, the clinical question and previous findings. Immunohistochemistry (IHC) was performed selectively; systematic screening for surface antigens potentially suitable for antibody–drug conjugates was not performed.

### Clinical evaluation and assessment of therapy

MoFa conferences took place every two weeks and included a faculty from the following departments: internal medicine III (hematology and oncology), internal medicine I (gastrointestinal oncology, endocrine tumors), institute of pathology, institute of human genetics, radiology as well as nuclear medicine (see workflow in Fig. 1a). Furthermore, medical experts from different clinics involved in the patients’ treatment participated.

MoFa decisions were based on the diagnostic reports of the contributing institutions and on coordinated levels of evidence implemented across all centers of personalized medicine (Supplementary Table S2).

All MoFa recommendations were stored in a structured manner in ULTIMA, the tumor board system in which all tumor boards of the University Hospital of Ulm are documented. Clinical outcome and imaging were reviewed at approximately three-month intervals after the start of MoFa-recommended therapy.

PFS1 was defined as the interval from the start of the immediately preceding systemic line to progression. PFS2 was defined as the interval from the start of the selected MoFa-guided treatment line to progression or death; patients without an event were censored at the date of their last adequate assessment. Index treatment had to be initiated by 31 December 2023, with PFS follow-up through 30 June 2024. The PFS2/PFS1 ratio was evaluated using a descriptive threshold of > 1.3 (adapted from Von Hoff et al. 2010); ratios based on censored PFS2 observations were considered lower bounds.

The ratio analysis included adults with interpretable consecutive systemic treatment lines. A preceding line stopped for toxicity rather than progression was considered ineligible for ratio analysis. Reasons for exclusions are detailed in Supplementary Table S5.

### Imaging

Additionally, we evaluated the radiological response in patients who had imaging shortly prior to the start of the recommended therapy and at least one study over eight weeks after therapy initiation. Radiologic response assessment was performed retrospectively based on available cross-sectional imaging in the clinical real-world setting. Patients were assessed in accordance with RECIST 1.1 criteria (Eisenhauer et al. 2009).

All measurements were taken using mint Lesion (Mint Medical GmbH, Heidelberg, Germany).

### Statistical analysis

OS was defined from first MoFa discussion to death from any cause in all groups. Patients who were alive at the end of follow-up were censored at the last documented date on which they were known to be alive, with follow-up administratively censored at 30 June 2025. Groups were defined according to treatment status on 31 December 2023: G1, recommendation implemented; G2, recommendation not implemented; G3, no recommendation.

Kaplan–Meier estimates were reported with Greenwood log–log 95% confidence intervals. Follow-up was estimated using the reverse Kaplan–Meier method (Schemper and Smith 1996), and months were calculated as days/30.4375. Exploratory pairwise log-rank tests were adjusted for multiple comparisons using Holm adjustment. Baseline characteristics were summarised descriptively and missing values were not imputed.

Post hoc restricted mean survival time (RMST) analyses were performed at 24 and 36 months based on the Kaplan–Meier estimates (Royston and Parmar 2013). Greenwood covariances were used to derive variances, nominal 95% confidence intervals and Wald tests. Holm adjustment was applied separately to the six pairwise comparisons and the two global tests. The 24- and 36-month time horizons were selected post hoc based on inspection of the observed survival data. Calculations were performed using Python with NumPy and SciPy.

During the preparation of this manuscript, ChatGPT (OpenAI) and the AI-based editor in Microsoft Word were used to support language and editorial revision and to assist with data analysis and the preparation of tables and figures. All outputs were reviewed and edited by the authors, who take full responsibility for the final content of the manuscript.

## Results

### Clinical characteristics

Overall, 125 patients with sarcoma were included in our study; there were 69 female and 56 male patients (see Fig. 1b). Among the 125 patients, 95 patients had soft tissue sarcoma (leiomyosarcomas, *n* = 25; sarcomas not otherwise specified (NOS), *n* = 23; synovial sarcomas, *n* = 8; desmoplastic small round cell tumors (DSRCT) (Gaidzik et al. 2024), *n* = 7; angiosarcomas, *n* = 6; liposarcomas, *n* = 5; pleomorphic sarcomas, *n* = 5; fibrosarcomas (Gaidzik et al. 2023), *n* = 5; rhabdomyosarcomas, *n* = 4; epithelioid sarcomas, *n* = 2; malignant peripheral nerve sheath tumors (MPNST), *n* = 2; alveolar soft part sarcoma (ASPS), *n* = 1; dermatofibrosarcoma, *n* = 1; clear cell sarcoma, *n* = 1) and 30 patients had bone sarcomas (Ewing sarcomas, *n* = 8; chondrosarcomas, *n* = 7; chordomas, *n* = 7; osteosarcomas (Seeling et al. 2025), *n* = 7; malignant giant cell neoplasia, *n* = 1).

The median recorded age at first MoFa discussion was 53 years (range, 6–83 years of age), with most patients aged 50–69 years (Fig. 1b). Patients included in the PFS2/PFS1 analysis had a median age of 55 years, compared with 52 years among the remaining 96 patients (Table 1; Supplementary Table S5).

**Table 1:** Characteristics of the sarcoma cohort and PFS analysis subsets

Characteristic	All (*n* = 125)	PFS evaluable (*n* = 29)	Other (*n* = 96)	Treated, PFS excluded (*n* = 12)
Recorded age, median (IQR), years	53 (37–63)	55 (40–65)	52 (35–61.25)	34 (25.25–60.75)
Recorded age, range, years	6–83	22–81	6–83	12–76
Female, n (%)	69 (55.2)	14 (48.3)	55 (57.3)	4 (33.3)
Male, n (%)	56 (44.8)	15 (51.7)	41 (42.7)	8 (66.7)
Soft tissue sarcoma, n	95	18	77	8
Bone sarcoma, n	30	11	19	4
Number of prior treatment lines, available, n	30	29	1	1
Number of previous lines, median of available counts	2	2	2	2

Baseline ECOG and metastatic status were not uniformly available. The 12 treated patients excluded from ratio analysis are included among the other 96 patients

Patients included in the 29-patient PFS2/PFS1 ratio cohort had received a median of two prior systemic treatment lines.

### Presentation in MoFa and molecular genetic findings

All 125 patients were presented in our MoFa at least one time. Based on the stage of disease, ECOG of the patients, previous therapies and already performed extended molecular diagnostics, the first recommendation was advanced molecular genetic testing by available NGS-DNA/RNA panels, preferentially on a new biopsy, or the last available tumor sample (see Fig. 1a). Molecular profiling comprised DNA panel sequencing in 105/125 (84.0%), Archer RNA sequencing in 48/125 (38.4%), OncoScan analysis in 42/125 (33.6%) and NCT MASTER profiling in 5/125 (4.0%). Twelve patients were not included in any of these profiling groups (Fig. 1d).

**Fig. 2:**
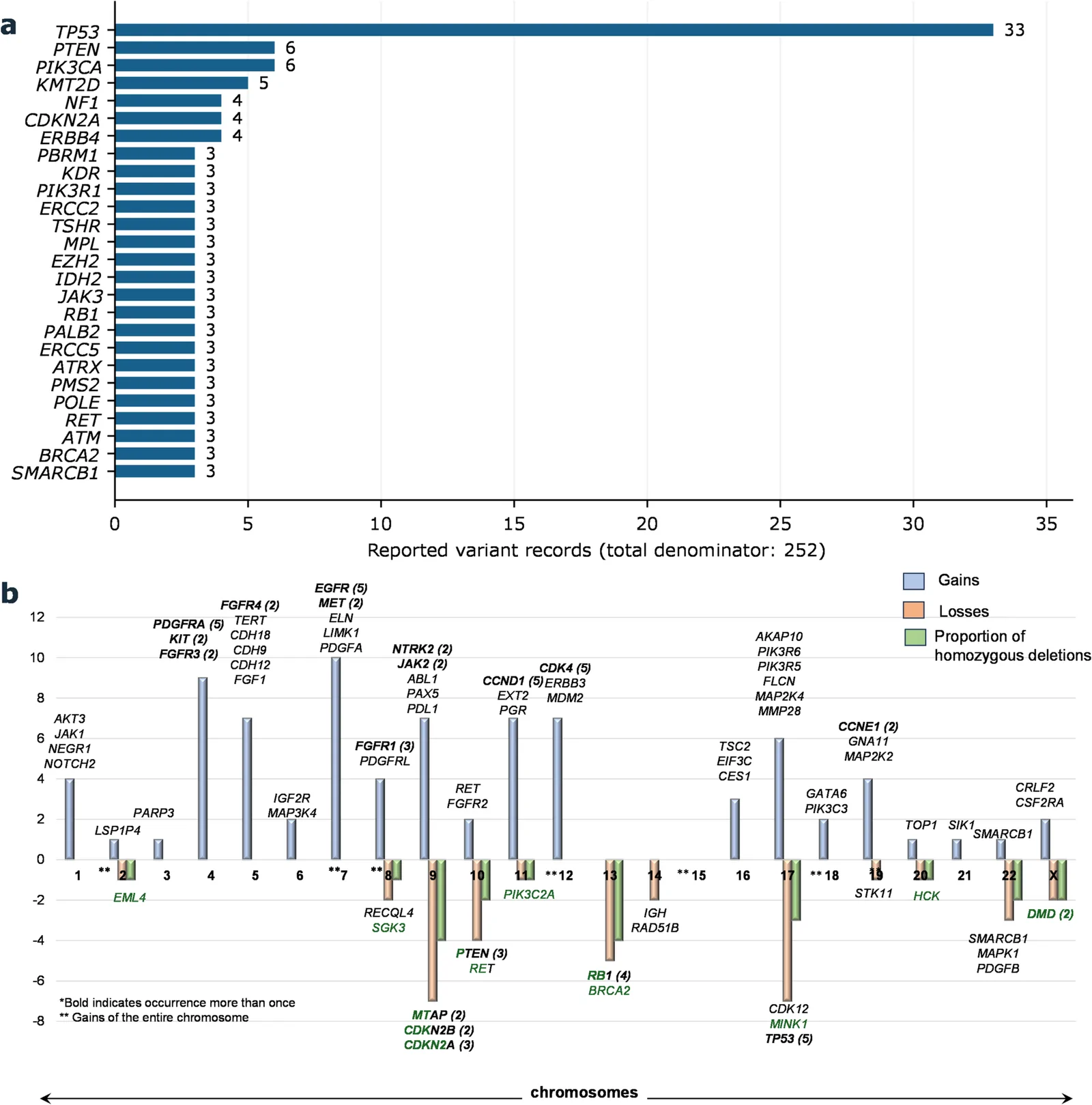
Molecular findings. **a**: Genes accounting for ≥ 1% of 252 variant records; counts describe variants, not patients. **b**: Copy-number findings in 42 patients. Gains are above and losses below the axis; green denotes homozygous losses or hemizygous *DMD* loss in males. Bold labels indicate recurrent findings; asterisks denote whole-chromosome gains

Overall, 252 pathogenic/likely pathogenic variant records (class 4 and class 5) were identified in 133 different genes by NGS. *TP53* was the most frequently altered gene, accounting for 13.1% of all pathogenic/likely pathogenic variants identified (*n* = 33), followed by alterations in the genes *PTEN* (*n* = 6), *PIK3CA* (*n* = 6), *KMT2D* (*n* = 5), *NF1* (*n* = 4), *ERBB4* (*n* = 4), *CDKN2A* (*n* = 4), *PBRM1* (*n* = 3), *KDR* (*n* = 3), *PIK3R1* (*n* = 3), *ERCC2* (*n* = 3), *TSHR* (*n* = 3), *MPL* (*n* = 3), *EZH2* (*n* = 3), *IDH2* (*n* = 3), *JAK3* (*n* = 3), *RB1* (*n* = 3), *PALB2* (*n* = 3), *ERCC5* (*n* = 3), *ATRX* (*n* = 3), *PMS2* (*n* = 3), *POLE* (*n* = 3), *RET* (*n* = 3), *ATM* (*n* = 3), *BRCA2* (*n* = 3), *SMARCB1* (*n* = 3) (see Fig. 2a).

Copy-number determination using OncoScan array analyses in 42 patients identified 117 gains or losses considered as potentially instructive for targeted treatment. The copy-number changes detected are shown in Fig. 2b. The genes most frequently affected or considered as potential targets for treatment were *TP53* (*n* = 5), *CDK4* (*n* = 5), *CCND1* (*n* = 5), *EGFR* (*n* = 5), *PDGFRA* (*n* = 5), followed by *RB1* (*n* = 4), *CDKN2A* (*n* = 3), *PTEN* (*n* = 3) and *FGFR1* (*n* = 3).

In our cohort, imbalance profiles suggesting homozygous losses occurred most frequently at the *RB1* locus, followed by *CDKN2A* and *TP53*; hemizygous losses affected *DMD* in male patients.

In addition, Archer RNA fusion panel analyses were performed in 48 of 125 patients (38.4%). The analysis failed in five patients and revealed no fusion in 34 patients. In the remaining nine cases, the findings included two *ESR1* exon 7 skipping events, one *TFG*::*ADGRG7* fusion, one *TES*::*MET* fusion, one *SS18*::*SSX2* fusion, one *EWSR1*::*FLI1* fusion, one *EWSR1*::*NFATC2* fusion, one *HNRNPC*::*NTRK1* fusion, and one *ASPSCR1*::*TFE3* fusion (Supplementary Table S3).

### Therapy recommendation and therapy evaluation

In 54 patients, no specific treatment recommendation could be made (Fig. 1c). Among the other 71/125 patients (56.8%) with a specific therapy recommendation, 30 did not receive treatment based on MoFa recommendations by 31 December 2023. Sixteen patients died before the recommended treatment could be initiated. Other reasons for non-implementation were surveillance (*n* = 3), continuing systemic therapy (*n* = 1), reimbursement pending or declined (*n* = 2 each), patient preference (*n* = 2), and clinical deterioration / poor clinical condition (*n* = 2). The reason was unknown in two patients (Supplementary Table S7).

The treatment recommendation was implemented in 41 patients (32.8%), corresponding to 57.7% of patients with a specific treatment recommendation. Of these, 29 patients were evaluable for PFS2/PFS1 analysis. Reasons for exclusion were pediatric age (*n* = 2), absence of prior systemic treatment (*n* = 2), non-comparable treatment intervals (*n* = 3), insufficient endpoint documentation or follow-up (*n* = 3), a concurrent primary malignancy (*n* = 1), and inadequate drug exposure (*n* = 1) (Supplementary Table S5).

### Radiographic evaluation of tumor response

RECIST 1.1-based radiographic assessment (Eisenhauer et al. 2009) was suitable in 14 patients, as suitable paired imaging was not available for all treated patients.

**Fig. 3:**
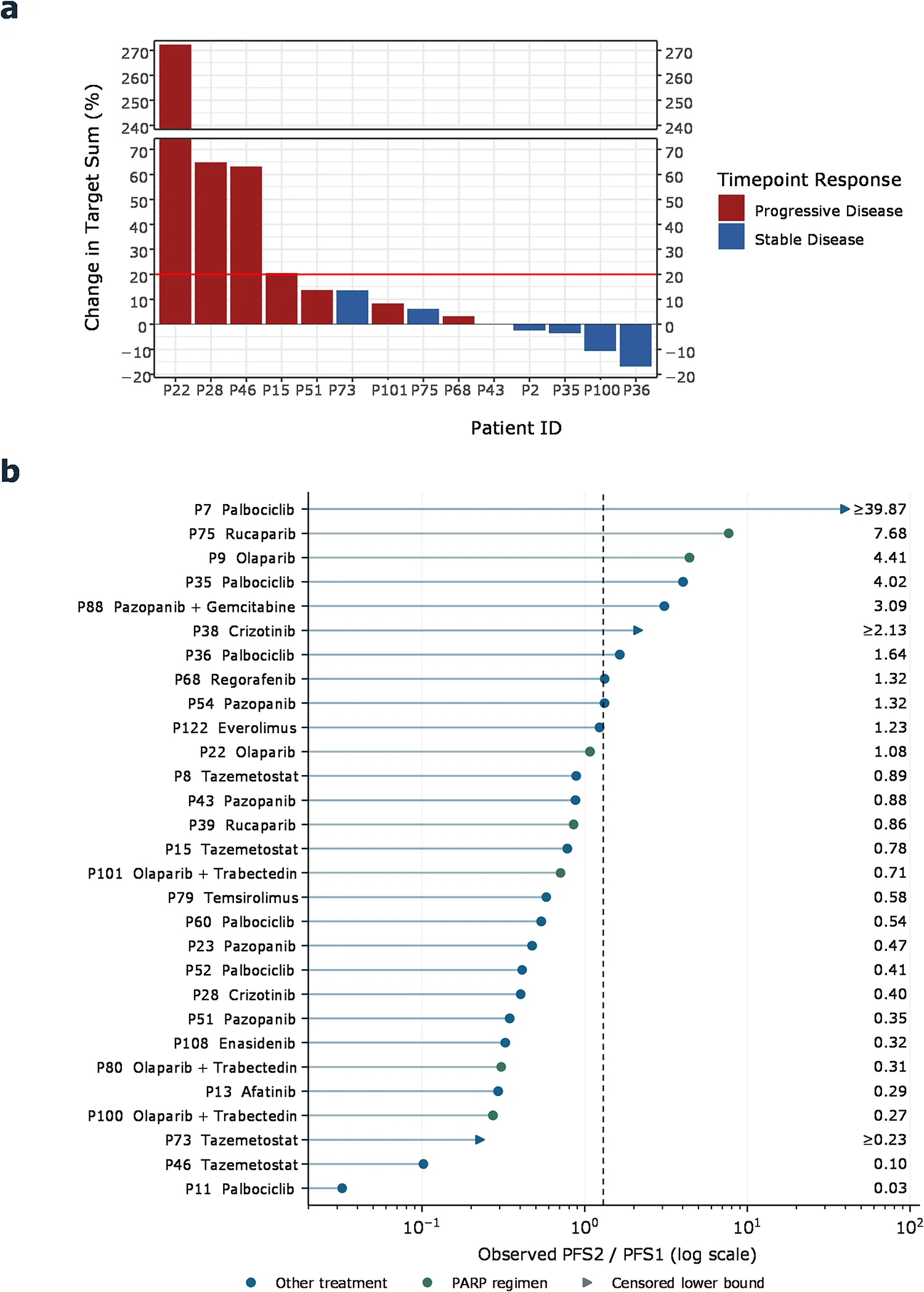
Response assessment and within-patient PFS ratio. **a**: RECIST assessment: Progressive disease (PD), *n* = 8 and stable disease (SD), *n* = 6. New lesions may establish PD despite < 20% target-lesion growth. **b**: PFS ratios in 29 patients; logarithmic axis; dashed line, 1.3; green, PARP-containing regimen. Arrowheads mark censored lower bounds in P7, P38 and P73

Among these 14 patients with RECIST 1.1-based assessment, eight (57%) were classified as having progressive disease (PD), while six (43%) exhibited stable disease (SD). Figure 3a summarizes the individual changes in tumor burden under therapy. Notably, progression according to RECIST 1.1 may be established by the appearance of new lesions, irrespective of whether the 20% increase in the sum of target lesion diameters required for progression based on target lesions alone has been reached (Eisenhauer et al. 2009).

### Outcome assessment based on PFS2/PFS1 Ratio as well as on overall survival (OS)

Progression-free survival under MoFa-guided therapy (PFS2) was compared with that on the immediately preceding systemic line (PFS1) in 29 of 41 treated patients for whom both intervals were evaluable (Von Hoff et al. 2010; Supplementary Table S4; Fig. 3b). Nine of 29 patients (31.0%) achieved a PFS2/PFS1 ratio > 1.3, whereas 19 experienced an event before reaching this threshold. PFS2 was censored for patients P7, P38, and P73. Median PFS2 was 102 days (95% CI, 63–233), compared with 150 days for median PFS1 (Supplementary Figs. S1–S2).

Four patients achieved a PFS2/PFS1 ratio > 4: P7 and P35 received palbociclib, P75 rucaparib and P9 olaparib. Among the seven patients treated with PARP inhibitors in the ratio cohort, two (P75 and P9) had a PFS2/PFS1 ratio > 1.3 (Supplementary Table S6).

**Fig. 4:**
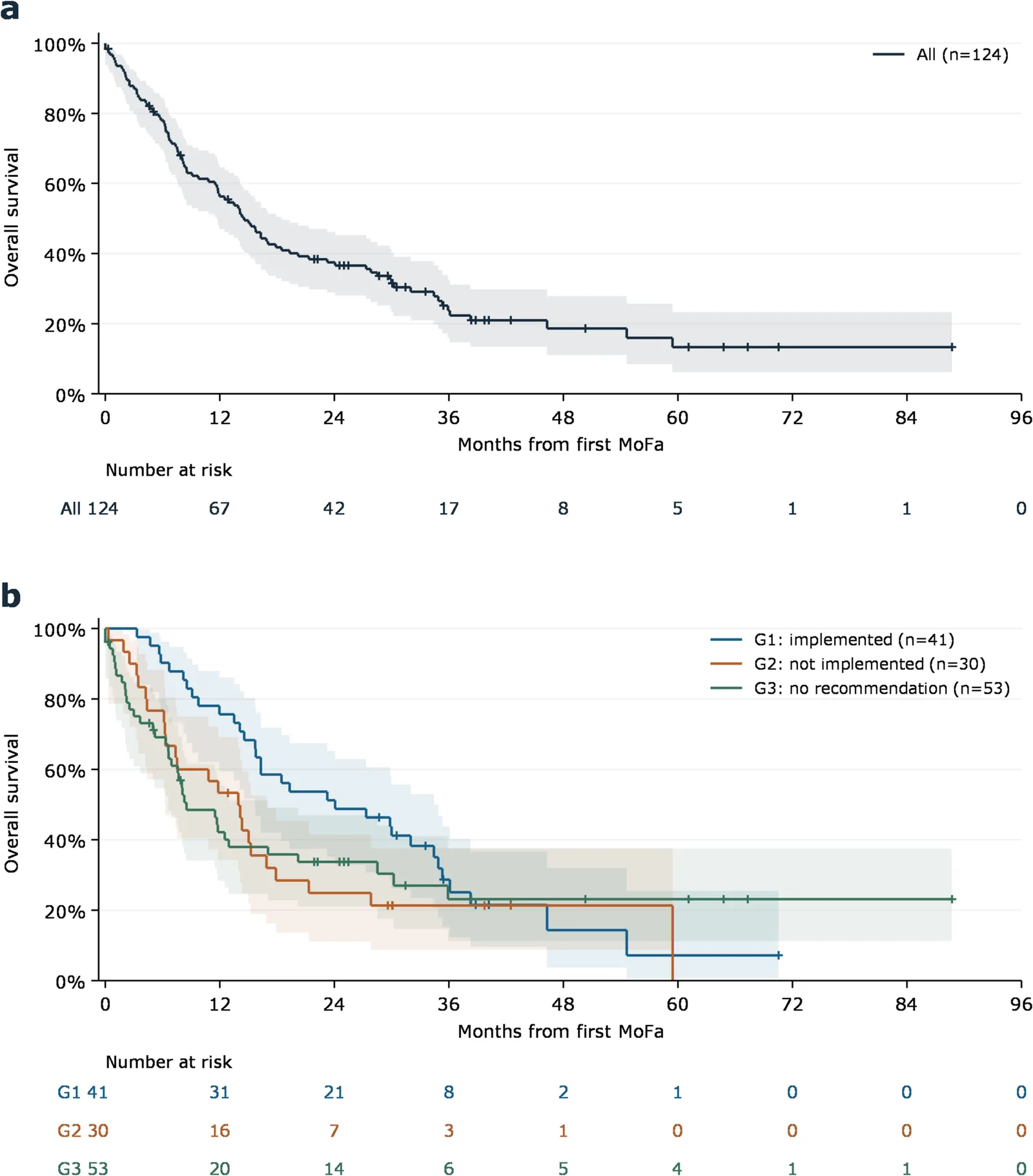
Overall survival from first MoFa discussion. **a**: OS in 124 patients: 92 deaths, 32 censored. **b**: Fixed groups G1–G3 (*n* = 41/30/53). Follow-up ended on 30 June 2025. Bands show 95% confidence intervals; crosses indicate censoring. Risk tables count patients observed immediately before each time. Global log-rank *p* = 0.220. Fixed-group comparisons retain immortal-time bias and confounding

OS was assessable in 124 of 125 patients, with 41, 30 and 53 patients in groups 1–3, respectively (Fig. 4). One patient had no interpretable survival interval from first MoFa discussion. During follow-up, 92 deaths and 32 censored observations were recorded. Median OS from first MoFa was 14.6 months (95% CI 11.7–18.5), with a reverse Kaplan–Meier median follow-up of 39.7 months (95% CI 31.4–50.3). By group, median OS was 24.1 months (95% CI 15.7–34.4) in group 1 compared to 13.9 months (6.3–16.9) in group 2 and 8.5 months (6.6–17.1) in group 3. Kaplan–Meier analysis showed no statistically significant difference among the three groups (log-rank *p* = 0.220); no pairwise comparison was statistically significant after Holm adjustment.

As a post hoc analysis, RMST at 24 months was 18.17, 13.09 and 12.32 months in G1–G3. The RMST differences were 5.08 months between groups 1 and 2 (nominal 95% CI, 1.45–8.70; Holm-adjusted *p* = 0.0303) and 5.85 months between groups 1 and 3 (nominal 95% CI, 2.49–9.21; Holm-adjusted *p* = 0.0038). RMST contrasts at 24 and 36 months are presented in Supplementary Fig. S3.

### Recurrent myxofibrosarcoma in a 26-Year-Old Patient with *BRCA2* deletion (Gaidzik et al. 2023)

**Fig. 5:**
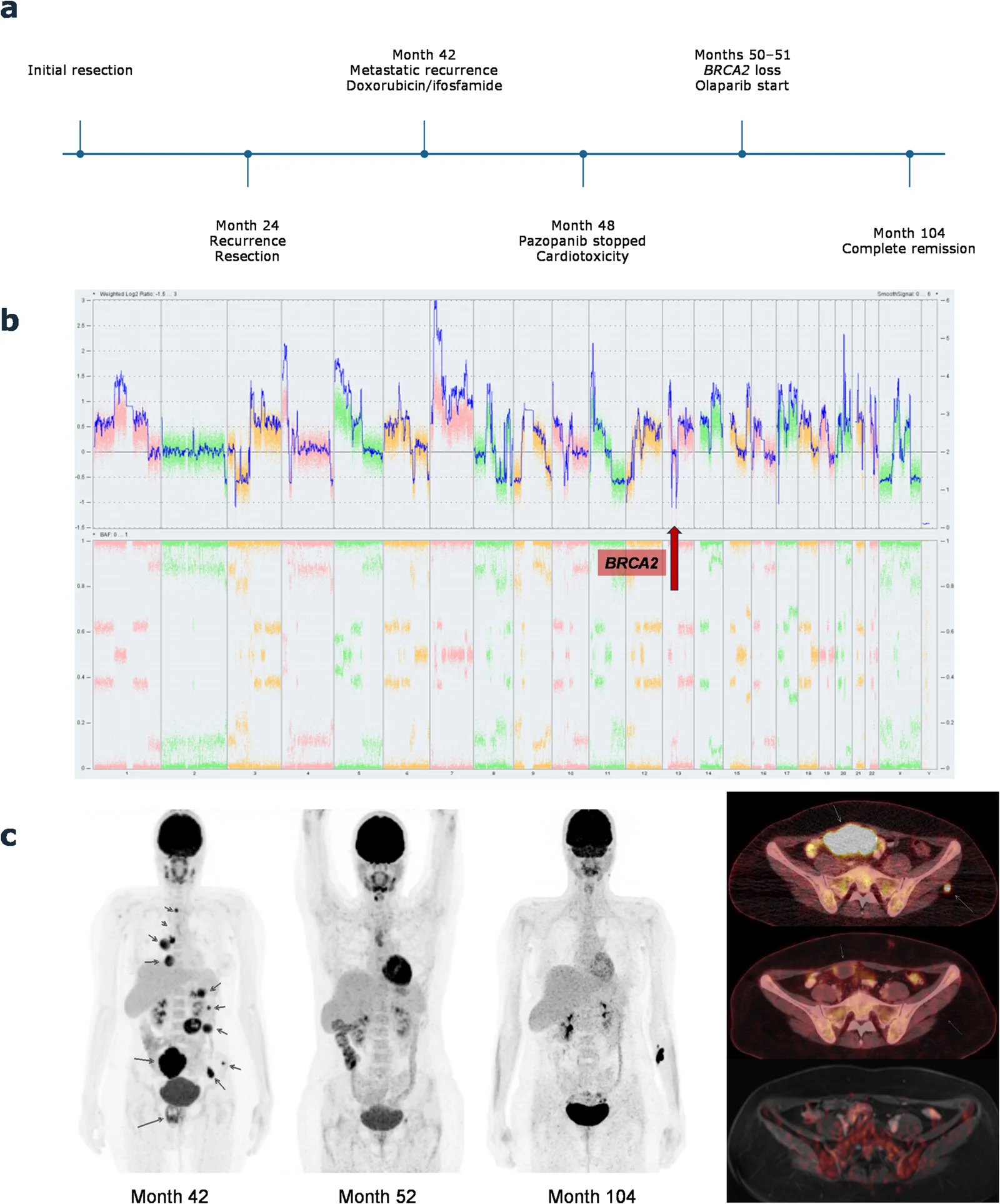
Clinical illustration: P2. **a**: P2 treatment course. Pazopanib stopped for cardiotoxicity; P2 is excluded from ratio analysis. Olaparib began at month 51; complete remission was documented at month 104. **b**: OncoScan showing likely homozygous *BRCA2* loss; germline status untested. **c**: PET/CT images. Extended individual follow-up is separate from the cohort PFS endpoint

As an illustrative example, patient P2 is presented here in more detail with extended follow-up (see Fig. 5a). A 26-year-old female patient initially underwent resection of a myxofibrosarcoma but experienced recurrence after 24 months. At the time of the first recurrence, it was evident that the tumor had become more aggressive as indicated by an increase in the Ki67 index from 20% to 40% and a worsening of histological grading (FNCLCC 3 + 3+1 = 7, compared to the initial 2 + 2+1 = 5). Following a second surgical resection, the patient experienced a second relapse at month 42, now with multiple metastases involving soft tissue, upper abdomen, lung, lymph nodes, bone, and pancreas. Concurrent with the recommendation of doxorubicin/ifosfamide, molecular genetic profiling was initiated through our MoFa, which revealed a *PDGFRB* (p.Asn666Lys, VAF 76%, class 4) variant. After six cycles of chemotherapy, the patient showed a PR, but residual fluorodeoxyglucose (FDG) activity remained on the PET scan. Pazopanib therapy was started but had to be discontinued due to significant heart failure in the first month of treatment (month 48).

OncoScan analysis (see Fig. 5b) identified a complex aberrant karyotype and a likely homozygous deletion of the *BRCA2* gene, which was not assessed in the germline. This molecular finding led to the rationale for switching to olaparib at month 51, which was well tolerated; complete remission was documented at month 104 (see Fig. 5c). This extended individual follow-up is separate from the cohort PFS endpoint.

## Discussion

Despite limited therapeutic progress in advanced sarcoma, molecular tumor boards such as our MoFa have emerged as valuable tools for individualized treatment decision-making, particularly in patients with limited standard treatment options (Horak et al. 2017). By integrating next-generation sequencing and other omics-based technologies, such platforms enable more precise molecular characterization and may support personalized therapeutic strategies (Horak et al. 2017, 2021; Berclaz et al. 2023). However, other MTB cohorts have reported challenges in treatment implementation and substantial heterogeneity in outcome definitions, limiting direct comparisons across studies (Heinrich et al. 2023; Gladstone et al. 2025).

Genomic studies of adult soft tissue sarcomas have described subtype-specific molecular alterations and frequent copy-number changes (The Cancer Genome Atlas Research Network 2017). TMB also varies across tumor types and sarcoma cohorts (Chalmers et al. 2017; Xu et al. 2020). We identified 252 pathogenic or likely pathogenic variant records; differences in molecular profiling and reporting preclude inference of TMB or patient-level mutation prevalence from this aggregate count.

While genomic profiling formed the basis of molecular characterization in our cohort, protein-level assessment was not performed systematically across the cohort. In particular, there was no predefined immunohistochemical screening for actionable surface antigens or potential targets for antibody–drug conjugates. Selective IHC cannot establish the prevalence of actionable antigens or the additional diagnostic and therapeutic yield of systematic antibody–drug conjugate screening. Standardized protein-level assessment may complement genomic profiling in future MTB workflows; however, its incremental diagnostic and therapeutic yield cannot be assessed from the present cohort.

All treatment recommendations were based on coordinated levels of evidence implemented across the Centres for Personalized Medicine and aligned with frameworks used by other precision oncology consortia (Horak et al. 2017, 2021; Supplementary Table S2).

The biological rationale underlying the treatment recommendations can be mainly categorized into several pathways, including cell cycle regulation (e.g. *CDK4*, *Cyclin D1*), epigenetic regulation (e.g. *EZH2*, *INI1*, *IDH2*), DNA repair and chromosomal stability (e.g. *BRCA2*, *ATM*, *CHEK2*), mTOR/PI3K/AKT (*PTEN*), as well as receptor tyrosine kinases (e.g. *PDGFR*, *VEGFR*, *FGFR2*). These pathways reflect established mechanisms in cancer biology and genomic medicine (Garraway and Lander 2013). MDM2 inhibition represents an additional molecularly defined therapeutic approach in liposarcoma and has been investigated in early-phase clinical trials (Gounder et al. 2023).

PARP inhibition exploits defects in DNA repair (Davar et al. 2012; Curtin and Szabo 2020), with early clinical activity demonstrated in *BRCA*-associated epithelial cancers (Fong et al. 2009). Two of seven PARP-treated patients had PFS2/PFS1 ratios > 1.3. Prolonged disease control in individual cases provides a hypothesis-generating signal supporting further prospective evaluation. However, the small number of patients and heterogeneity of the underlying molecular alterations require cautious interpretation. In addition, high VAF alone does not establish biallelic loss, and functional HRD assays were not performed in this cohort.

Implementation of molecularly guided treatment recommendations was limited by death, clinical deterioration, access restrictions and patient preference, while the reasons for non-implementation remained unknown in two cases. These findings highlight the importance of identifying molecularly guided treatment options while patients can still receive treatment, although our data do not allow an assessment of whether earlier molecular profiling translates into improved treatment access or clinical outcomes. Furthermore, the regulatory process of obtaining reimbursement approval needs to be considered when implementing off-label treatment strategies.

Radiologic response could be assessed in a subset of our sarcoma patients using RECIST 1.1 criteria (Eisenhauer et al. 2009). In a retrospective series of patients with sarcoma treated with trabectedin, RECIST and Choi classifications were largely concordant although discordant responses were observed in individual cases (Ceddia et al. 2024). Incomplete paired imaging limits interpretation and generalizability of the radiographic response findings.

Median OS was longer among patients in whom the molecularly guided treatment recommendation was implemented, although the global log-rank test did not reach statistical significance. The exploratory RMST identified differences between groups that may warrant further prospective evaluation. Interpretation is limited by substantial potential for immortal-time bias, as patients had to survive and remain clinically fit until treatment initiation, as well as by treatment selection and unmeasured confounding. Additional limitations include the retrospective design and the heterogeneity of diagnoses, molecular alterations, and treatment strategies. Performance status is an established prognostic factor in advanced sarcoma (Iwai et al. 2023), but standardized baseline ECOG performance status data were not uniformly available in our cohort.

Our real-world experience suggests that targeted sequencing panels may represent a pragmatic initial approach to molecular profiling in advanced sarcoma. Whole-genome sequencing can provide additional diagnostic and potentially therapeutic information (Watkins et al. 2024); however, the present data do not establish an optimal molecular profiling strategy.

## Supplementary Information

Below is the link to the electronic supplementary material.

Supplementary Figure S1

Supplementary Figure S2

Supplementary Figure S3

Supplementary Table S1

Supplementary Table S2

Supplementary Table S3

Supplementary Table S4

Supplementary Table S5

Supplementary Table S6

Supplementary Table S7

## Data Availability

Data are unavailable due to privacy or ethical restrictions.
