# Supplementary material for: Molecular tumor board in advanced sarcoma: treatment implementation and clinical outcomes in a real-world cohort
Source: J Cancer Res Clin Oncol. 2026 Oct 22;152(10):201. doi: 10.1007/s00432-026-06651-1 (PMC13691023; doi:10.1007/s00432-026-06651-1)
Supplement: Supplementary file 2 [file 432_2026_6651_MOESM2_ESM.pdf]

Supplementary Figure S2

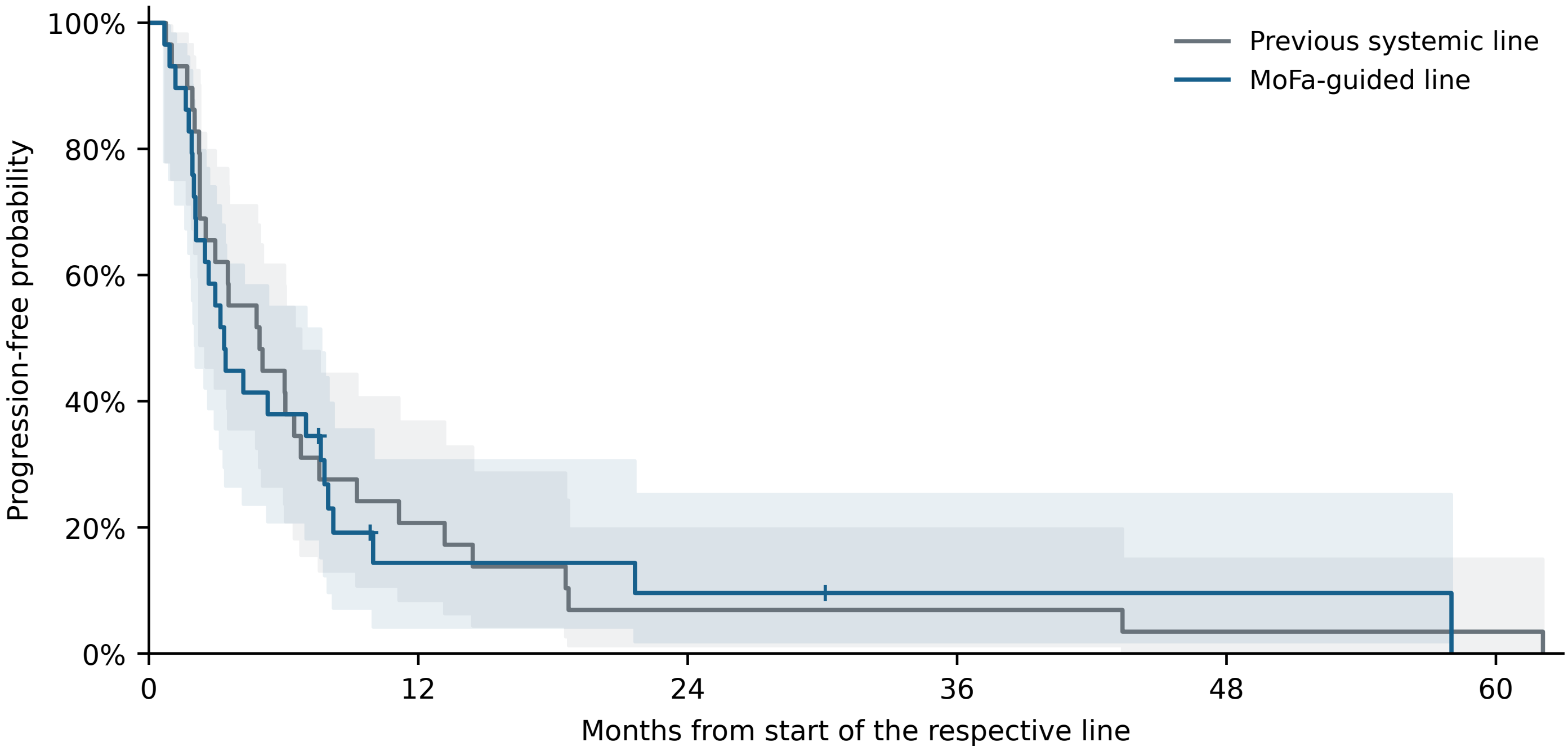

Number at risk

	0	12	24	36	48	60
PFS1	29	6	2	2	1	1
PFS2	29	3	2	1	1	0

Paired-line PFS curves with Greenwood log-log 95% confidence intervals and censoring marks. PFS1 and PFS2 concern the same 29 patients and are presented descriptively; no independent-sample comparison is implied. Risk tables show patients under observation immediately before each time.

Molecular Tumor Board in Advanced Sarcoma: Treatment Implementation and Clinical Outcomes in a Real-World Cohort

Journal of Cancer Research and Clinical Oncology

Christoph Weinfurtner, Moritz Kaupp-Merkle, Ralf Marienfeld, Uwe Gerstenmaier, Jakob Niewöhner, Tobias Kolb, Axel Fürstberger, Sonja Dahlum, Paul Herzog, Ambros J. Beer, Regine Mayer-Steinacker, Meinrad Beer, Thomas F.E. Barth, Thomas Seufferlein, Nadine T. Gaisa, Mathias Wittau, Stephan Stilgenbauer, Alexandra von Baer, Markus Schultheiß, Reiner Siebert, Wolfgang Thaiss, Hartmut Döhner, Verena I. Gaidzik

Corresponding author: Verena I. Gaidzik, Department of Internal Medicine III, University Hospital Ulm, Albert-Einstein-Allee 23, 89081 Ulm, Germany. Email: verena.gaidzik@uniklinik-ulm.de
