# Supplementary material for: Molecular tumor board in advanced sarcoma: treatment implementation and clinical outcomes in a real-world cohort
Source: J Cancer Res Clin Oncol. 2026 Oct 22;152(10):201. doi: 10.1007/s00432-026-06651-1 (PMC13691023; doi:10.1007/s00432-026-06651-1)
Supplement: Supplementary file 4 [file 432_2026_6651_MOESM5_ESM.pdf]

Supplementary Table S2. Classification scheme developed for the molecular tumor boards of the National Center for Tumor Diseases (NCT) and the German Cancer Consortium (DKTK) (Leichsenring et al. 2019).

**Same entity**	M1A	Predictive value of the biomarker or clinical effectiveness of the corresponding drug in a molecularly stratified cohort was demonstrated in a prospective study or a meta-analysis in the same tumor type.
	M1B	Predictive value of the biomarker or clinical effectiveness of the drug in a molecularly stratified cohort was demonstrated in a retrospective cohort or case-control study in the same tumor type.
	M1C	Case study or single unusual responder indicates the biomarker is associated with response to the drug in the same tumor type.
**Different entity**	M2A	Predictive value of the biomarker or clinical effectiveness of the corresponding drug in a molecularly stratified cohort was demonstrated in a prospective study or a meta-analysis in a different tumor type.
	M2B	Predictive value of the biomarker or clinical effectiveness of the drug in a molecularly stratified cohort was demonstrated in a retrospective cohort or case-control study in a different tumor type.
	M2C	Case study or single unusual responder indicates the biomarker is associated with response to the drug in a different tumor type.
**Preclinical**	M3	Preclinical data (in vitro, in vivo models or functional genomics studies) demonstrate that the biomarker predicts response to a specific drug treatment, supported by scientific rationale.
**Biological rationale**	M4	Biological rationale that associates the biomarker with altered signaling pathway activity or drug sensitivity without direct clinical or preclinical evidence for response to the drug.

**Additional Modifiers:**	
is	several in situ data and studies on patient material (e.g. IHC, FISH) support the biomarker and the level of evidence.
iv	in vitro data
Z	Drug is approved for use with the specific biomarker (Z= EMA approval, Z(FDA)=FDA approval)
R	Biomarker predicts resistance to the drug.

Bibliography

(1) Leichsenring J, Horak P, Kreutzfeldt S, et al (2019) Variant classification in precision oncology. Int J Cancer 145:2996–3010. https://doi.org/10.1002/ijc.32358

Molecular Tumor Board in Advanced Sarcoma: Treatment Implementation and Clinical Outcomes in a Real-World Cohort

Journal of Cancer Research and Clinical Oncology

Christoph Weinfurtner et al.
Corresponding author: Verena I. Gaidzik, Department of Internal Medicine III, University Hospital Ulm, Albert-Einstein-Allee 23, 89081 Ulm, Germany. Email: verena.gaidzik@uniklinik-ulm.de
